# Eye Tracking-Enhanced Deep Learning for Medical Image Analysis: A Systematic Review on Data Efficiency, Interpretability, and Multimodal Integration

**DOI:** 10.3390/bioengineering12090954

**Published:** 2025-09-05

**Authors:** Jiangxia Duan, Meiwei Zhang, Minghui Song, Xiaopan Xu, Hongbing Lu

**Affiliations:** 1 Department of Military Biomedical Engineering, Air Force Medical University, No. 169 Changle West Road, Xi’an 710032, China; duanjx@fmmu.edu.cn (J.D.); 15653811835@163.com (M.S.); 2 Shaanxi Provincial Key Laboratory of Bioelectromagnetic Detection and Intelligent Perception, No. 169 Changle West Road, Xi’an 710032, China; 3 College of Electrical Engineering, Chongqing University, No. 174, Shazheng Street, Shapingba District, Chongqing 400000, China; zhangmw_play@163.com

**Keywords:** eye tracking, deep learning, medical image analysis, data efficiency, interpretability, multimodal integration

## Abstract

Deep learning (DL) has revolutionized medical image analysis (MIA), enabling early anomaly detection, precise lesion segmentation, and automated disease classification. However, its clinical integration faces two major challenges: reliance on limited, narrowly annotated datasets that inadequately capture real-world patient diversity, and the inherent “black-box” nature of DL decision-making, which complicates physician scrutiny and accountability. Eye tracking (ET) technology offers a transformative solution by capturing radiologists’ gaze patterns to generate supervisory signals. These signals enhance DL models through two key mechanisms: providing weak supervision to improve feature recognition and diagnostic accuracy, particularly when labeled data are scarce, and enabling direct comparison between machine and human attention to bridge interpretability gaps and build clinician trust. This approach also extends effectively to multimodal learning models (MLMs) and vision–language models (VLMs), supporting the alignment of machine reasoning with clinical expertise by grounding visual observations in diagnostic context, refining attention mechanisms, and validating complex decision pathways. Conducted in accordance with the PRISMA statement and registered in PROSPERO (ID: CRD42024569630), this review synthesizes state-of-the-art strategies for ET-DL integration. We further propose a unified framework in which ET innovatively serves as a data efficiency optimizer, a model interpretability validator, and a multimodal alignment supervisor. This framework paves the way for clinician-centered AI systems that prioritize verifiable reasoning, seamless workflow integration, and intelligible performance, thereby addressing key implementation barriers and outlining a path for future clinical deployment.

## 1. Introduction

Deep Learning (DL) has emerged as the cornerstone of modern artificial intelligence (AI) in medical image analysis (MIA), demonstrating exceptional performance in critical clinical tasks including precise anatomical segmentation, early lesion identification, and diagnostic classification—fundamentally enhancing diagnostic accuracy and workflow efficiency [1]. While these advances enable more precise, data-driven medicine, the ‘black box’ nature of DL models creates persistent challenges in results interpretability and consistent performance [2], namely the critical barriers to full clinical applications [3].

A fundamental limitation of current DL approaches lies in their purely data-driven nature, which often diverges from human clinical reasoning. While DL excels at learning hierarchical patterns [4,5,6], medical imaging datasets are frequently inadequate, leading to spurious correlations and algorithmic biases that compromise both generalizability and interpretability. Recent solutions focus on integrating clinical expertise, either by incorporating physician knowledge to augment limited training data [7] or by correcting shortcut learning through medical priors [8], effectively bridging AI with human intelligence to improve robustness. One promising approach in this direction is the use of eye tracking (ET) technology [9]. ET technology is a mature, non-invasive method with three decades of research, providing unique insights into medical decision-making processes. By capturing clinicians’ gaze patterns during image/text review [10,11], ET analysis provided valuable insights into an AI’s usability for MIA [12]. ET-derived attention data enhance DL models by achieving the following: (i) guiding feature selection toward clinically relevant regions, (ii) reducing reliance on spurious features (mitigating overfitting) [9,13], and (iii) improving generalization. For example, Mall et al. [14] improved CNN performance in mammography by incorporating radiologists’ visual search patterns [15]. Beyond accuracy improvements, ET enhances model interpretability by revealing how experts integrate multimodal information (imaging findings, clinical knowledge, and visual cues). This facilitates human-centered AI development [16] by enabling multimodal architectures to combine data-driven learning with clinical reasoning [17]. Specifically, ET data can serve as an additional input modality [18], directing models to diagnostically relevant areas and advancing both multimodal learning models (MLMs) [19] and vision–language models (VLMs) [20].

**Figure 1:**
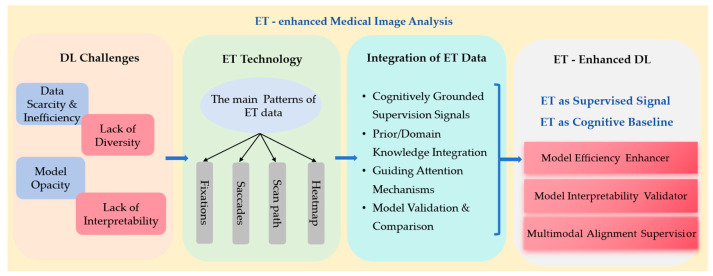
The overall framework of this review.

While existing reviews on ET in MIA provide comprehensive coverage of hardware and model performance [17,21], they critically overlook two key areas: (i) ET’s role in enhancing model interpretability, and (ii) its potential in emerging MLMs and VLMs. Our work systematically bridges these gaps by presenting a unified framework and research roadmap that demonstrates ET’s transformative potential for medical AI. Key contributions of this review are as follows:

• A systematic review with a generalized framework. We present the first comprehensive review of ET-enhanced MIA, introducing a generalized framework where ET data plays three critical roles, including data efficiency enhancers (reducing annotation dependency), interpretability validators (aligning AI attention with clinician reasoning), and multimodal alignment supervisors (bridging visual and textual domain information in image analysis). Figure 1 illustrates this taxonomy and situates an intuitive overview of the challenges of data scarcity and model opacity faced in DL in the domain of MIA. By integrating different ET data patterns with the DL model, it highlights the three important roles that ET plays in enhancing the DL model.

• Structured Analysis of ET Integration in MIA. Through rigorous evaluation of over 100 studies, we provide the first unified analysis of how ET data: (i) guide clinically relevant feature extraction; (ii) validate model interpretability via gaze-aligned saliency maps; (iii) optimize model performance with human attention priors; and (iv) enable multimodal fusion in MLMs/VLMs.

• Translational Roadmap. Based on emerging trends and critical gaps in the field revealed by structured synthesis, we further identify key challenges in clinical deployment, propose feasible solutions for real-world clinical application, and suggest future directions for ET-enabled medical AI.

Overall, this research introduces a novel framework integrating ET data to enhance data efficiency, interpretability, and multimodal alignment in MIA; provides the first unified analysis of ET data’s role in guiding feature extraction, validating interpretability, optimizing performance, and enabling multimodal fusion; and offers a comprehensive roadmap addressing clinical deployment challenges and proposing solutions for real-world application, highlighting future directions for ET-enabled medical AI.

## 2. Methods

### 2.1. Eligibility Criteria

This systematic review strictly adheres to the Preferred Reporting Items for Systematic Reviews and Meta-Analyses (PRISMA) [22] guidelines to ensure methodological rigor in the literature synthesis. The review protocol was registered in the International Prospective Register of Systematic Reviews [23] (PROSPERO; ID: CRD420251117996). Ethics approval is not necessary for this review. The inclusion criteria prioritized studies employing DL architectures with integrated ET modalities for medical image interpretation.

### 2.2. Search Strategy and Literature Selection

This study evaluates how integrating ET data with DL enhances MIA. Using PRISMA guidelines, we established inclusion (IC) and exclusion criteria (EC) for paper selection, ensuring a transparent screening process that identifies relevant studies while filtering out unsuitable ones. Table 1 specifies inclusion and exclusion criteria that enforce methodological rigor.

### 2.3. Data Extraction and Quantitative Synthesis

To support our qualitative analysis, we summarized the specific performance metrics reported in the included studies, such as AUC, F1-score, precision, recall, and false-positive rates. A formal meta-analysis with pooled effect sizes was not possible because the studies varied too widely in their imaging tasks, model types, datasets, and evaluation methods. Instead, we selected one key metric from each study. We prioritized the main test set result or the most clinically meaningful outcome. We then grouped these results by their task, whether it was segmentation, detection, or classification. For studies that reported relative changes, like improvements over a baseline, we used those figures directly. This narrative synthesis approach is considered a best practice when high variability among studies prevents statistical pooling.

### 2.4. ET Data Quality and Consistency

ET acquisition methods vary considerably across studies, which has significant implications for gaze precision, robustness to head motion, and downstream model performance. The studies reviewed generally employ three main types of eye-tracking devices: (i) remote eye trackers [24], (ii) eye-tracking glasses [25], and (iii) webcam-based trackers [26]. Data obtained from these devices vary considerably across several dimensions, including sampling rate, calibration protocol, head stabilization strategy, precision and drift correction methods, data loss rate, and the process of registering gaze to the image. Emerging research demonstrates that well-engineered webcam-based methods can achieve accuracy approaching laboratory standards—particularly when integrated with rigorous calibration and quality control—thereby facilitating large-scale studies. These considerations motivate our recommendation for standardized reporting and benchmarking using shared resources to improve consistency across different acquisition setups.

## 3. Results

**Figure 2:**
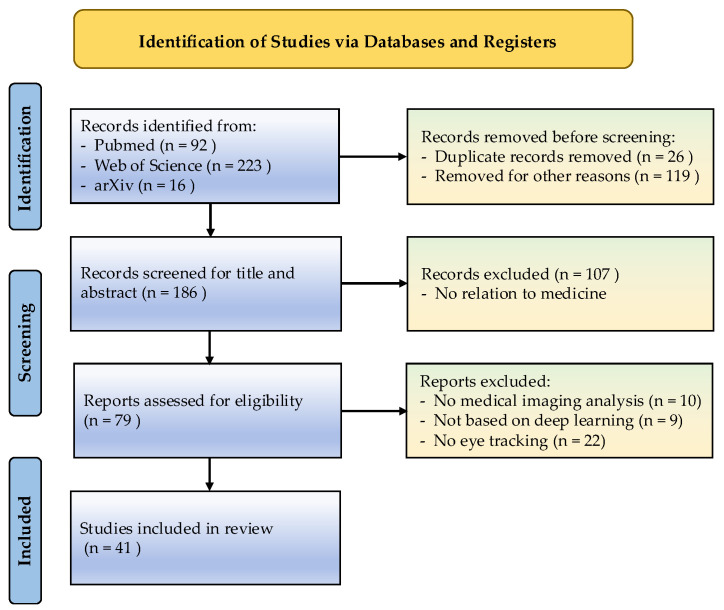
Flow diagram of the review process using modified PRISMA.

We conducted a systematic literature search across PubMed, Web of Science, and arXiv (January 2020–April 2025) using the Boolean query ((“eye tracking” OR “gaze*”) AND (“medical*” OR “medical image*” OR “radiology” OR “CAD” OR “computer-aided diagnosis”) AND (“deep learning” OR “DL” OR “CNN” OR “DNN”)) with an English-language restriction. The search yielded 331 initial records (Web of Science: 223; PubMed: 92; arXiv: 16). Of 331 initially identified records, 41 studies met all inclusion criteria following deduplication and a two-stage screening process. This involved (i) abstract-level exclusion of non-ET, non-medical, or non-DL studies and (ii) full-text review for relevance (see PRISMA diagram, Figure 2). Supplementary records were identified through backward reference searching and preprint screening.

**Figure 3:**
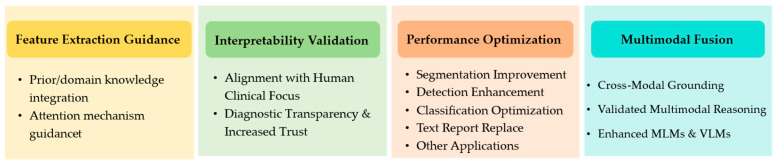
Overview of the different aspects covered in this review.

Through systematic analysis of the final paper corpus, we identified the following four key research dimensions, each substantiated by case studies and empirical evidence: (a) Feature Extraction Guidance- ET data address critical challenges in MIA (e.g., feature redundancy and salient pattern identification with limited data) by directing feature extraction to clinically relevant regions; (b) Interpretability Validation- comparative analysis of model attention maps with expert ET patterns establishes decision alignment, bridging AI outputs with human cognitive processes for transparent interpretability; (c) Performance Optimization- ET-enhanced DL models demonstrate improved accuracy and robustness, particularly in data-scarce scenarios; and (d) Multimodal Fusion—emerging applications integrate ET with MLMs and VLMs to refine medical image–text grounding and cross-modal attention mechanisms. Figure 3 provides an overview of these interrelated dimensions and their applications in medical AI. It groups evidence into feature extraction guidance, interpretability validation, performance optimization and multimodal fusion.

### 3.1. ET Data and Patterns Used

In medical image processing, ET devices (e.g., eye trackers) are employed to record physicians’ gaze positions, scan paths, and dwell times during image interpretation in real-time. These visual attention metrics are subsequently transformed into gaze heatmaps or fixation sequences, serving as “human attention labels” for DL models. Commonly utilized ET patterns include fixations, saccades, scan paths, and heatmaps. As summarized in Table 2, typical application scenarios consistently revolve around the core concept of “transforming physicians’ authentic visual attention into algorithmically utilizable supervisory signals.” Table 2 maps common ET patterns to mechanisms and representative use cases. This evolution demonstrates how ET data are transitioning from being merely an “observation tool” to assuming multifaceted roles as “supervisory signals, alignment bridges, and interpretability aids” in medical image processing, encompassing segmentation, alignment, modeling, interpretation, etc.

### 3.2. Commonly Used ET for Feature Extraction

#### 3.2.1. Effective Feature Extraction Is Fundamental to DL Models

While effective feature extraction is crucial for enabling DL models to capture discriminative patterns [31] and enhance both performance and generalizability [32], current approaches exhibit significant limitations in medical imaging applications. Recent advances include the following: Yan et al. [33] combining spatial and channel attention mechanisms for improved bronchoscopic image analysis; Sarkar et al. [34] developing hybrid frameworks using pre-trained ResNet-18 features; Muksimula et al. [35] integrating dense neural networks with connection-wise attention for multiscale MRI feature extraction; and Inception ResNetv2-based approaches for cervical cancer detection [36].

However, these methods share two critical shortcomings: (i) failure to incorporate clinical prior knowledge (e.g., anatomical constraints or radiologist expertise), and (ii) limited capacity for precise lesion localization due to insufficient medical-domain feature extraction. These limitations fundamentally constrain diagnostic accuracy in complex clinical scenarios.

#### 3.2.2. ET Used to Guide the Feature Extraction of DL

**Figure 4:**
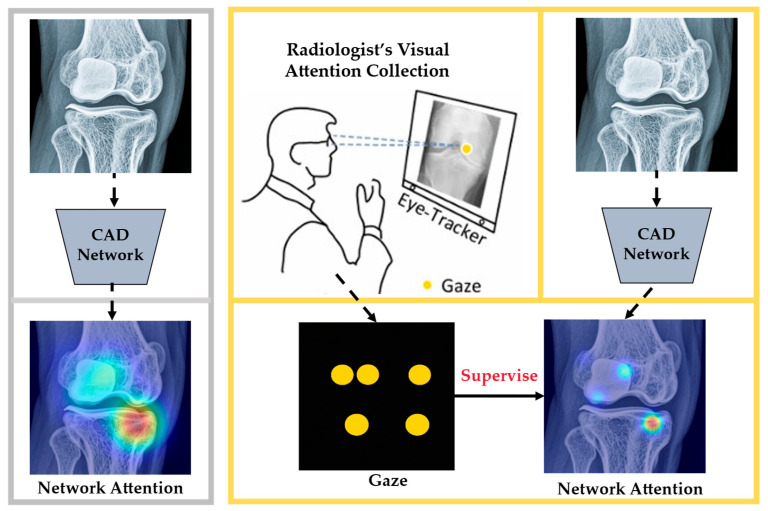
Unlike the conventional method (left panel), the right panel employs ET data to supervise the network’s attention mechanism, enhancing classification accuracy and abnormality localization performance. Adapted from Wang et al. (2022) [39].

ET data quantify visual behavior through fixation points, durations, saccades, and scan paths, revealing expert attention patterns. Table 3 organizes representative uses of ET for feature extraction and distills outcome-oriented highlights. Kok et al. [37,38] demonstrated that radiologists’ systematic visual search strategies enhance DL feature extraction when incorporated as prior knowledge. ET-guided attention mechanisms dynamically weight image regions using radiologists’ diagnostic focus areas, improving clinical feature recognition accuracy. Optimal integration is achieved via two primary methodologies:

Prior/domain knowledge integration. ET data are typically converted into attention heatmaps and integrated with image data either as training inputs or supervisory signals. This integration enables DL models to more precisely localize task-relevant features and develop more discriminative representations. Several studies demonstrate this approach’s effectiveness, e.g., Wang et al. [39] constrained model attention using gaze-derived attention maps, mimicking human visual prioritization as illustrated in Figure 4. The schematic contrasts baseline and gaze-supervised training, highlighting improved localization and classification when gaze constrains attention. Jiang et al. [40] developed a gaze-guided attention module that fuses fixation maps with fundus images, significantly improving lesion feature extraction and classification accuracy in diabetic retinopathy CAD systems. Dmitriev et al. [41] quantitatively compared radiologists’ gaze patterns with CNN activation maps to identify diagnostically relevant features.

Recent advances demonstrate ET’s clinical versatility: Franceschiello et al. [42] established ET as a biomarker for neurological assessment, Moinak et al. [43] integrated gaze with radiomics (GazeRadar) to enhance chest X-ray localization, while Xie et al. [27] and Wang et al. [44] leveraged gaze-supervised learning and augmentation (GCA) to improve breast cancer detection—collectively proving ET’s value as prior knowledge for diagnostic feature learning.

Attention mechanism guidance. ET data further optimize feature extraction in attention-based architectures (e.g., transformers or self-attention), explicitly demonstrating feature importance during decision-making [45]. This approach has become increasingly prominent in medical imaging analysis [46,47,48]. Kong et al. [49] proposed gaze-guided detection transformer (Gaze-DETR), a detection transformer that integrates gaze data to enhance feature extraction and reduce false positives in vulvovaginal candidiasis diagnosis. Bhattacharya et al. [50] introduced RadioTransformer, which utilizes radiologists’ gaze patterns to model visual–cognitive behavior for chest radiograph diagnosis. These innovations demonstrate how ET data refine attention mechanisms, bridging human expertise and computational models.

**Table 3:** The typical application of ET used for feature extraction.

Ref.	ET Patterns	Feature Extraction Method	Imaging Modality (Diseases)	DL Models	Observations and Highlights
[40]	Fixation distribution	Weighted fixation maps served as an auxiliary imaging modality (concatenated with fundus images) and as supervised masks to guide feature extraction.	Fundus images (diabetic retinopathy)	ResNet-18	Using weighted fixation maps as auxiliary masks yielded the best performance, with an accuracy of 73.50% and an F1-score of 77.63%, confirming that gaze-guided feature extraction benefits diabetic retinopathy recognition.
[44]	Heatmaps	A saliency prediction model mimics radiologist-level visual attention, and the predicted gaze heatmap conditions positive pair generation via GCA, preserving critical information like abnormal areas in contrastive views.	Knee X-ray (knee osteoarthritis)	ResNet-50	Predicted gaze heatmaps used in gaze-conditioned augmentation raised knee-OA classification accuracy from 55.31% to 58.81%, indicating that expert–attention-conditioned views outperform handcrafted augmentations.
[49]	Heatmaps and scan paths	Expert fixation heatmaps and scan path vectors are encoded as sparse attention weights, concatenated with the original image features along the channel dimension, and then fed into the model for joint feature extraction.	Colposcopy images (vulvovaginal candidiasis)	Gaze-DETR	Encoding expert heatmaps and scan paths as sparse attention improved detection: average precision increased across thresholds, recall reached 0.988, and false positives declined.

These approaches show that radiologists’ gaze patterns provide valuable guidance for DL models, significantly enhancing feature extraction effectiveness when integrated into diagnostic models.

### 3.3. ET Used to Validate the Interpretability of DL

#### 3.3.1. Interpretability of DL in MIA

Recent advances in DL have significantly enhanced medical imaging analysis, improving diagnostic accuracy and optimizing clinical workflows. Although DL models achieve exceptional performance through complex, multi-layered architectures with numerous parameters—often characterized as “black box” systems [51]—their decision-making processes remain opaque due to high-level abstractions [52]. This inherent trade-off between interpretability and performance represents a fundamental challenge in medical AI research [53].

Explainable artificial intelligence (XAI) has emerged as a critical attribute of AI technology, directly influencing the acceptance and adoption of AI tools in clinical practice [54]. By providing transparent insights into a model’s architecture and decision logic [55], interpretability fosters trust in AI systems, revealing how internal components interact to generate outputs. As a key research frontier in DL, interpretability enhances model transparency, facilitates clinical adoption by building user trust, and enables performance optimization through explainable reasoning [56,57,58].

In radiology AI, interpretability serves three essential functions: (i) strengthening clinician confidence in high-stakes diagnostic decisions, (ii) improving diagnostic efficiency and accuracy, and (iii) identifying model limitations for iterative refinement [59]. Despite substantial accuracy improvements in DL-based computer-aided diagnosis (CAD) systems, the persistent lack of interpretability remains a major barrier to widespread clinical implementation [59].

#### 3.3.2. Interpretability Methods in DL

Saliency maps (SMs) are a key tool for interpreting DL model decisions in medical imaging. They address the following two critical questions: (1) Which input features most influence model outputs? and (2) Where does the model focus when making decisions? By quantifying output sensitivity to input features, SMs visualize the most influential image regions for predictions, thereby revealing critical input–output relationships. Common methods of SM analysis include saliency [60], gradient-weighted class activation mapping (GradCAM) [61], SmoothGrad [62], gradient SHAP (Grad SHAP) [63], integrated gradients [64], etc.

SMs visually highlight the regions and features most influential in model predictions. This enables users to identify the input features driving model decisions, evaluate whether the model utilizes clinically relevant features, and enhance transparency in the decision-making process. However, current interpretability methods still face some key limitations. For instance, techniques like Grad-CAM and SmoothGrad are primarily designed for CNN and often fail to precisely localize subtle diagnostic features [59]. Most critically, existing interpretability approaches often misalign with medical professionals’ cognitive processes, which rely heavily on clinical expertise and visual pattern recognition when analyzing medical images.

#### 3.3.3. Application of ET in Validating Interpretability

ET technology has emerged as a well-established methodology for investigating radiologists’ visual attention patterns, offering valuable insights into diagnostic reasoning processes and decision-making strategies [21]. Empirical studies demonstrate a significant correlation between fixation characteristics and clinical expertise, with experienced radiologists exhibiting more efficient visual search patterns characterized by focused attention on diagnostically relevant regions and substantially reduced dwell time on non-informative areas [65]. The non-invasive nature of ET enables capture of clinicians’ natural viewing behavior without disrupting diagnostic workflows, presenting unique advantages for CAD system development. Recent advances have successfully incorporated ET data to align computational attention mechanisms with radiologists’ gaze patterns, simultaneously improving diagnostic performance and model interpretability [13]. This synergistic approach demonstrates particular potential for enhancing model robustness and accelerating the translation of research findings into clinical practice.

ET data provide valuable insights into radiologists’ diagnostic focus areas, enabling direct comparison between DL model decisions and expert reasoning. Strong alignment between model saliency maps (SMs) and ET heatmaps validates prediction interpretability, while discrepancies reveal optimization opportunities. Recent advances include Gaze Estimation Models (GEMs) [66], which use context-aware networks to simulate radiologists’ complete image analysis, capturing both visual attention and cognitive patterns. Kim et al. [67] systematically compared DL-generated SMs with ET data in chest X-ray classification, finding significantly better spatial alignment with expert gaze patterns for correct versus incorrect predictions across six saliency methods. Khosravan et al. [68] introduced a collaborative CAD (C–CAD) system to leverage ET data of screening MR images in improving interpretability. In the detection of pulmonary nodules, the doctor’s attention is often focused on the edge and morphological features of the nodules. Aresta et al. [69] utilized gaze information for automatic lung nodule detection interpretations. By comparing the attention distribution of a DL model with the doctor’s ET map, it is possible to verify that the model is focusing on the right areas. If the two are highly consistent, the model’s decision is reliable. If they are inconsistent, the model may need to be adjusted. This consistency analysis helps to enhance the interpretability of the model, which not only makes the model more transparent but also provides doctors with more trust basis, making the decision-making process of the model more transparent and credible. Wang et al. [39] confirmed ET supervision improves CAD interpretability. Ma et al. [30] developed eye gaze guided vision transformer (EG-ViT) incorporating radiologists’ domain knowledge through gaze-guided mask generation and mask-driven visual attention, which enhanced decision transparency on Inbreast [70] and SIIM-ACR [71] datasets and harmful shortcut learning rectified by eye gaze guidance conspicuously. The EGMA framework enhances medical image–text understanding through cross-modal mapping, demonstrating that even limited ET data can significantly boost multimodal processing and model interpretability [28].

These studies collectively establish a framework for clinically aligned AI development, where ET data serve dual critical functions as supervisory guidance during model training and a validation benchmark for decision verification. Through precise alignment of computational attention patterns with radiologists’ visual search strategies, ET technology effectively bridges the conceptual divide between artificial decision-making and clinical reasoning processes. This integration significantly enhances both the perceived trustworthiness and measurable interpretability of CAD systems in medical practice.

### 3.4. ET Used to Improve the Performance of DL

ET technology captures clinicians’ visual cognition, integrating this expertise into DL models to enhance performance and streamline clinical workflows through human–AI synergy.

#### 3.4.1. ET Used as Prior Knowledge to Improve the Performance of DL

DL models learn statistical patterns, while radiologists’ gaze tracks clinically meaningful features. ET quantifies these expert attention patterns through SMs, providing supervision to align deep neural networks (DNNs) with clinical reasoning to enhance performance. Recent studies confirm ET-DL integration significantly improves MIA. Table 4 surveys performance-oriented ET–DL studies and summarizes their practical gains as concise highlights.

Segmentation improvement. Stember et al. [72] employed ET data to generate attention heatmaps, which were aligned with model SMs to optimize parameter adjustment toward diagnostically relevant regions, thereby improving segmentation accuracy. Additionally, they integrated ET with speech recognition to annotate MRI brain images, achieving 92% accuracy in training lesion marking and 85% in independent testing [73]. Gaze2Segment [74] automated breast CT scan segmentation with ET guidance. Xie et al. [27] developed a direct ET integration method that outperforms Gaze2Segment in robustness and adaptability. Their approach enhances performance in limited-data scenarios, demonstrates resilience to erroneous gaze patterns, and achieves superior segmentation accuracy for classes with significant variations compared to state-of-the-art (SOTA) models. The GazeSAM system [75] combines ET with the segment anything model (SAM) to support gaze-guided, interactive segmentation to boost segmentation accuracy and efficiency.

Detection enhancement. The GCA [44] model significantly improved the performance of contrastive learning methods on knee X-ray images. While the gaze-guided detection transformer (Gaze-DETR) [49] model integrated ET data to reduce false positives, the comprehensive tests confirm that Gaze-DETR surpasses existing leading methods, showcasing remarkable improvements in the detection accuracy of vulvovaginal candidiasis and the generalizability of the model. Colonnese et al. [76] use ET data to identify autism spectrum disorder (ASD)-related gaze behaviors, outperforming benchmarks. Karargyris et al. [77] generate AI training datasets, and Tian et al. [78] localize glaucoma features in OCT reports, collectively advancing diagnostic accuracy through gaze-guided AI. Ma et al. [9] introduced a saliency-guided vision transformer (SGT) to suppress shortcut learning by incorporating artificial prior knowledge. This knowledge was derived from SMs predicted by an ET-trained model, enabling the SGT to process images without requiring actual ET data during inference.

Classification optimization. Huang et al. [79] leveraged ET data to improve MIA with limited training data. By introducing a novel auxiliary attention block (AAB), they achieved strong performance in 3D tumor segmentation and 2D chest X-ray classification. Similarly, Zhu et al. [80] describes a gaze-guided class activation mapping (GG-CAM) method to directly regulate the formation of network attention based on radiologists’ visual attention for the chest X-ray pathology classification problem, which remains challenging.

Text report replacement. Zhao et al. [81] proposed Medical Contrastive Gaze Image Pretraining (McGIP) to address scarce radiology reports by using radiologists’ fixation patterns to identify similar image pairs (positive pairs) for contrastive learning. Evaluated on Tufts Dental [82] and Inbreast [70], McGIP demonstrates strong plug-and-play potential for clinical contrastive learning frameworks.

**Table 4:** Summary of ET-DL research works to improve performance.

Task/Domain	Reference	Gaze Processing	Year	Datasets	Type of Disease	DL Model	Highlights
Segmentation	Stember et al. [72]	ET mask	2019	Images from PubMed and Google images	meningioma	U-net [13]	ET-derived masks improved meningioma segmentation performance.
	Stember et al. [73]	Gaze position/Fixation	2021	BraTS [83]	brain tumor	CNN models [84]	Gaze supervision enabled accurate brain tumor labeling and boosted downstream accuracy.
	Xie et al. [27]	Fixation heatmaps	2024	Inbreast [27]	breast cancer	U-net [13]	Gaze-supervised segmentation increased Dice and mIoU under limited data.
Detection	Wang et al. [44]	Fixation heatmaps	2023	Knee X-ray images [85]	osteoarthritis	U-net [13]	Gaze-conditioned contrastive views increased knee X-ray detection accuracy.
	Kong et al. [49]	Fixation heatmaps	2024	Private *	vulvovaginal candidiasis	Transformer [27]	Gaze-guided DETR raised AP/AR and reduced false positives in VVC screening.
	Wang et al. [75]	Fixation Points	2023	GrabCut dataset [86] and Berkeley dataset [87]	abdomen disease	SAM [88]	Eye gaze with SAM improved interactive segmentation mIoU.
	Colonnese et al. [76]	Fixation Points	2024	“Saliency4ASD” dataset [89]	ASD	RM3ASD [90], STAR-FC [91], AttBasedNet [92], and Gaze-Based Autism Classifier (GBAC) [76]	Gaze features improved ASD classification across accuracy, recall, and F1-score.
	Tian et al. [78]	Fixation Points	2024	Private *	glaucoma	U-net [13]	Expert gaze guided OCT localization with higher precision, recall, and F1-score.
Classification	Huang et al. [79]	Fixation heatmaps	2021	BraTS [83] and the MIMIC-CXR-gaze [77]	brain tumor and chest disease	U-net [13], nnUnet [93], and DMFNet [94]	Gaze-aware attention strengthened segmentation/classification metrics with scarce data.
	Ma et al. [9]	Fixation heatmaps	2022	Inbreast [70] and SIIM-ACR [71]	chest disease	ResNet [95], Swin Transformer [96], and EfficientNet [96]	Gaze-predicted saliency curtailed shortcut learning and improved ACC, F1-score, and AUC.
	Zhu et al. [80]	Fixation heatmaps	2022	The multi-modal CXR dataset [77]	heart disease and chest disease	ResNet [95] and Efficient Net [97]	Gaze-guided attention raised precision and recall in chest X-ray classification.
Text report replacement	Zhao et al. [81]	Fixation heatmaps	2024	Inbreast [70] and Tufts dental dataset [70]	breast and dental diseases	ResNet [95]	Gaze-driven contrastive pretraining improved accuracy and AUC without text reports.

* Authors collected their ET data.

#### 3.4.2. Other Applications of ET in Enhancing MIA

Medical image Annotation

DNNs demand extensive labeled training data, whose manual annotation is costly and time-intensive [40]. ET technology offers an efficient alternative by passively capturing radiologists’ diagnostic attention patterns during routine readings. These gaze patterns naturally highlight anatomically and pathologically relevant regions, enabling automatic extraction of high-quality labels for DNN training. ET-based annotation frameworks match manual labeling accuracy while being faster and more clinically relevant [98]. For instance, Stember et al. [72] used ET to train models for segmentation, achieving parity with manual masks. Their subsequent work [73] combined ET with speech recognition to automatically label brain tumors. ET-generated heatmaps have also served as labeled data for model training and classification of Alzheimer’s [99].

Data Augmentation

ET data enhance DL by guiding clinically meaningful data augmentation. While traditional augmentation (e.g., rotation, scaling, cropping) improves model generalization, ET integration preserves diagnostically critical regions by aligning transformations with radiologists’ ET patterns [44]. This approach maintains lesion integrity while expanding dataset diversity [30,77]. For medical image classification, ET-driven augmentation systematically generates large-scale training sets that retain clinical relevance through attention-aware transformations [99].

To orient readers across heterogeneous settings, Table 5 compiles representative quantitative outcomes by task, reporting metrics exactly as stated in the source studies and highlighting where ET either improved accuracy or reduced errors. Examples include gains with Gaze-DETR in colposcopy, reductions of mammography false positives with RadioTransformer, and high accuracy for ET-assisted lesion annotation on brain MRI images. Table 5 reports representative quantitative outcomes by task to enable quick comparison.

### 3.5. ET in MLMs and VLMs

Multimodal data fusion seeks to exploit the complementary, cooperative, and redundant features of different modalities to aid in the diagnostic process [100]. ET data enhance medical multimodal learning by improving cross-modal alignment (e.g., image–text fusion) and feature representation through supervised training, while also validating model interpretability via ET-guided reasoning analysis [101,102].

#### 3.5.1. Cross-Modal Alignment

Recent studies using ET data during radiology report reading reveal how gaze patterns create explicit links between text passages and corresponding image regions. This helps MLMs learn better grounded representations and improves tasks like report generation or visual question answering [103]. Mention techniques like using ET maps as attention targets or incorporating ET coordinates into the model’s input [104].

**Figure 5:**
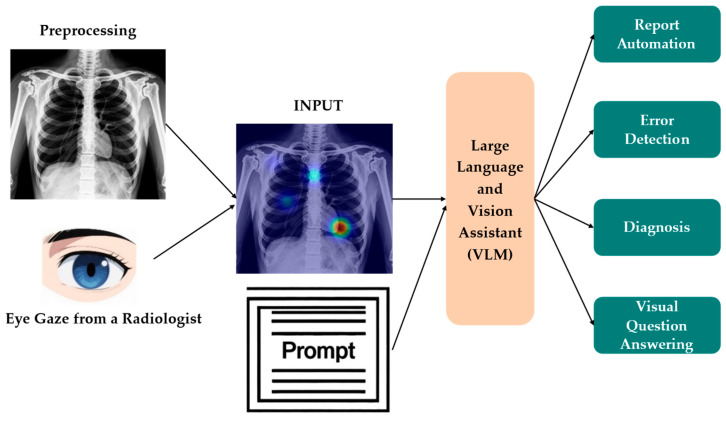
Overview of enhancing human–computer interaction in chest X-ray analysis using VLM with eye gaze patterns. Adapted from Kim et al. (2024) [20].

Early multimodal pre-training relied on millions of aligned image and text pairs, but in radiology and other domains such scale is unattainable; recent work shows that human ET data bridge this gap by providing pixel-level links between what experts read and where they look. The EGMA framework [28] effectively aligns images with corresponding text features to significantly enhance both the model’s multimodal processing capabilities and cross-modal feature fusion, particularly in zero-shot classification and retrieval tasks, and demonstrates a higher area under the ROC curve (AUROC) and enhanced image–text retrieval accuracy. Eye gaze guided cross-modal alignment network (EGGCA-Net) [105] incorporated eye gaze regions into a dual-branch encoder; aligning sentence prototypes with gaze-conditioned visual features reduces false findings in report generation, demonstrating ET data’s auxiliary role in aligning medical images with text to enhance feature extraction. Kim et al. [20] developed VLMs incorporating ET data with textual prompts to enhance feature extraction accuracy and zero-shot classification in chest X-rays, as illustrated in Figure 5. The pipeline overlays gaze on chest X-rays to steer the vision–language model’s attention during analysis.

Existing resources like the REFLACX [106] and MIMIC-Eye [107] datasets provide synchronized gaze-dictation pairs for chest X-rays, enabling supervised grounding benchmarks and ET-guided attention training. These studies show explicit ET supervision enhances text–image alignment, improving report generation fidelity while reducing annotation costs.

#### 3.5.2. ET-Supervised Training and Representation Learning

ET patterns act as a form of self-supervised yet highly informative guidance for representation learning [108]. Gaze pre-training (GzPT) [109] treats images with similar radiologist scan paths as positive pairs and introduces the Temporal Image Moment Analysis (TIMA) algorithm—a unified gaze similarity evaluation method adaptable to various image modalities (e.g., mammograms and dental X-rays) and gaze data formats (sequences and heatmaps), overcoming McGIP’s need for modality-specific metrics. FocusContrast [110] modals radiologists’ visual attention during X-ray diagnosis, predicting their gaze patterns on new images to guide attention-aware augmentation, ensuring disease-related abnormalities are prioritized. As a plug-and-play and framework-agnostic module, it consistently boosts SOTA contrastive learning methods in classification accuracy on knee X-ray datasets.

#### 3.5.3. Interpretability and Validation of Multimodal Reasoning

MLMs often act as more complex ‘black boxes’ versus single-modality systems. Researchers evaluate their interpretability by comparing model attention maps with human ET data [111]. Strong alignment boosts trust in model reasoning, while discrepancies may reveal spurious correlations [112]. This method provides unique insights into cross-modal reasoning beyond conventional validation.

Human gaze comparison offers a robust validation method for MLM reasoning [113]; the ‘Seeing Eye to AI’ approach measures how closely transformer attention matches human eye movements in video memorability tasks, showing strongest agreement for highly memorable content, and indicating models focus on the same key areas as humans when making confident predictions. Awasthi et al. [19] propose temporally grounded intention detection (TGID), which aligns multimodal model attention with radiologists’ reasoning by regressing ET heatmaps to intent captions. It detects shortcut learning via gaze–attention divergence, offers cross-modal validation, and builds clinical trust by spotting spurious correlations early. As summarized in Table 6, ET strengthens multimodal learning by improving alignment, pretraining efficacy, and reasoning transparency across methods.

## 4. Discussion

This review documents the transformative potential of ET-enhanced DL paradigms in MIA, demonstrating its dual capacity to address the following two fundamental limitations: (i) data inefficiency in model training and (ii) opacity in decision-making. By encoding radiologists’ visual attention patterns, ET delivers cognitively grounded supervisory signals that simultaneously improve learning efficiency in data-scarce scenarios, enhance model performance through clinically relevant feature prioritization, and establish interpretable decision pathways aligned with medical reasoning. Empirical evidence across segmentation, detection, classification, and report-generation tasks consistently demonstrates that ET-guided models improve diagnostic accuracy, mitigate overfitting, and align SMs with clinically pertinent image regions.

Although many studies report that ET enhances learning signals and decision alignment, the evidence is not uniformly positive. First, performance gains are often task- and domain-specific. For instance, despite designing attention-based CAD systems using expert gaze, Wang et al. [39] and Karargyris et al. [77] reported limited accuracy improvements over strong baselines. Second, gaze-attention alignment is not guaranteed; comparisons between radiologist eye movements and DL saliency maps often reveal imperfect correspondence. This indicates that gaze may highlight clinically relevant context unused by the model, or conversely, that model attention may capture discriminative cues outside of overt radiologist fixations. Third, gaze data quality (sampling rate, calibration error, drift, and data loss) and registration errors can inject noise that diminishes benefits or even harms training signals. Finally, architectural mismatch (e.g., naively injecting heatmaps into late layers) can underuse gaze information or over-constrain attention. Together, these mixed findings motivate stronger data-quality control, ablations (with/without ET), and domain-shift evaluations when claiming ET-related gains.

ET reinforces trust in AI-assisted decisions while extending these benefits to MLMs and VLMs through improved cross-modal alignment and refined reasoning processes, thereby signaling a paradigm shift toward clinician-centered, explainable medical AI. However, despite the proven value of radiologists’ ET data in validating and optimizing decision-making, key challenges persist in fully integrating this technology into DL systems.

Challenges and Future Trends: The integration of ET into DL pipelines shows considerable promise in MIA, yet it confronts three interrelated challenges. A primary obstacle is the difficulty of acquiring synchronized, high-precision ET data alongside medical images, a process that is both labor-intensive and costly. This has resulted in a scarcity of publicly available datasets, especially compared to those in natural image domains. Current resources are limited to repositories such as REFLACX [106] and TDD [82], and the expense of high-precision tracking equipment further restricts accessibility [114]. To mitigate data scarcity, transfer learning offers a viable approach [68,115]. It enables the application of knowledge derived from limited ET datasets collected in specific diagnostic contexts, such as lung cancer detection, to related domains like prostate cancer screening, thereby enhancing data utilization efficiency. Moreover, in scenarios where exact gaze localization is non-critical, low-cost webcam-based trackers provide a practical alternative for expanding data collection, despite their lower spatial resolution [26,116]. Second, consistent performance improvements are limited by the cognitive variability in ET data and the inherent complexity of its processing. ET recordings are high-dimensional and heterogeneous, requiring integration with task-specific objectives, contextual image information, and expert cognitive models to produce meaningful interpretations. Empirical studies indicate that simply incorporating ET data does not reliably improve model accuracy or sensitivity compared to baseline systems [39,77]. Nevertheless, the growing adoption of modern AI architectures, especially MLMs and VLMs, enables effective use of ET data within a weak supervision framework. These approaches allow ET information to guide attention mechanisms, anchor textual descriptions to relevant image regions, and offer implicit cues regarding feature importance during model training. By leveraging the representational capacity of large-scale models, this strategy presents a promising path to mitigate issues of data scarcity and expert bias. Consequently, ET integration is emerging as a crucial direction for building robust, interpretable, and clinically relevant medical AI systems. Third, issues of data quality, bias, and noise present considerable challenges. ET recordings are inherently susceptible to environmental influences, calibration inaccuracies, sensor noise, and variability across experts’ cognitive processes, which undermine their reliability as an objective ground truth [117]. To tackle these concerns, implementing comprehensive quality control measures is crucial. Such measures encompass advanced noise-filtering algorithms, rigorous calibration protocols, standardized acquisition environments and procedures, consensus-driven region definitions, multi-expert annotation systems, and extensive user training [21,117,118,119,120]. Furthermore, developing effective model-learning strategies is necessary to optimally utilize noisy or sparse ET signals, thus alleviating the drawbacks of simplistic integration approaches [74].

Moreover, reliable generalization and faithful reproducibility in studies that integrate eye tracking with deep learning depend on transparent reporting, deterministic data handling, and evaluation that extends beyond a single site or cohort. To enable meaningful comparison across studies, investigators should disclose the device used for gaze acquisition, the sampling rate, the calibration targets and repetitions, the method used for head stabilization, the mapping accuracy expressed in degrees of visual angle, the approach to drift correction, the proportion of missing samples, and the exact procedure by which gaze is registered to images. Reproducible practice further requires the release of code for preprocessing, quality control, and model training, together with fixed data partitions and random seeds, and with grouping at the patient level so that leakage is avoided. Claims about the contribution of eye tracking ought to be supported by paired experiments that hold the architecture and data constant while comparing training with and without gaze, and by stress tests that examine transfer across sites or across modalities. In addition to headline accuracy, researchers should make available interpretable artifacts such as representative gaze maps, model attention maps, and quantitative alignment summaries so that readers can evaluate the decision process and not only the final score. Whenever possible, work should be anchored on public resources such as REFLACX and MIMIC Eye so that results can be replicated exactly and compared fairly. We also encourage future studies to adopt explicit reporting and sharing practices to strengthen external validity.

In contrast to earlier reviews, our synthesis integrates a concise quantitative summary with a taxonomy of data quality and reporting recommendations, thereby enabling like-for-like comparisons across heterogeneous studies. This approach underscores the value of ET not only as a means of enhancing performance but also as a facilitator of reproducibility and a bridge between algorithmic attention and clinical reasoning.

## 5. Conclusions

ET characteristic analysis provides an objective and quantifiable tool for the field of medical imaging, capable of capturing the dynamic behaviors of radiologists in real-time during visual search, information extraction, and decision-making processes. By analyzing key parameters such as fixations, heatmaps, saccades, and scan paths, this method reveals the spatial distribution of visual attention and underlying cognitive mechanisms, thereby contributing to improved diagnostic accuracy and a deeper understanding of professional knowledge and models. With the iterative upgrading of lightweight, high-precision, non-invasive eye trackers and the maturation of data analysis algorithms, the role of eye-tracking technology in MIA is set to become increasingly prominent. ET technology provides a vital cognitive bridge between radiologists’ expertise and artificial intelligence, offering an inherently interpretable signal to guide next-generation multimodal systems. This integration enables AI that simultaneously achieves diagnostic-grade accuracy, transparent decision-making, data-efficient learning, and alignment with clinical reasoning patterns. To realize this potential, the following three critical pathways require focused development: (i) affordable tracking solutions (webcam/headset-based systems) for scalable deployment; (ii) large-scale, publicly available ET-image benchmark datasets are urgently needed to enable rigorous, reproducible evaluation of ET-guided models and privacy-preserving computational architectures for sensitive gaze data; and (iii) seamless workflow integration enabling real-time ET-guided clinical diagnostic assistance. By consolidating evidence across tasks and modalities and by specifying transparent reporting practices, this review provides a practical foundation for reproducible ET-enhanced medical AI. We anticipate that this framework will accelerate rigorous clinical translation where performance gains, interpretability, and external validity are advanced in tandem.

## Figures and Tables

**Table 1:** The outline of inclusion and exclusion criteria defined for this review.

List of Inclusion and Exclusion Criteria			
Inclusion Criteria (IC)		Exclusion Criteria (EC)	
IC1	Should contain at least one of the keywords.	EC1	Manuscripts containing duplicated passages lack originality or fail to contribute meaningful insights.
IC2	Must be sourced from reputable academic databases, such as PubMed and Web of Science.	EC2	The full-text publication could not be retrieved or accessed through available channels.
IC3	Published after 2019 (inclusive).	EC3	Study was rejected or contains a warning.
IC4	Publications included peer-reviewed journal papers, conference or workshop papers, non-peer-reviewed papers, and preprints.	EC4	Non-English documents or translations that exhibit structural disorganization, ambiguous phrasing, or critical information gaps.
IC5	Selected studies must demonstrate a clear alignment with DL and ET’s focus on medical image analysis, with relevant titles, abstracts, and content.	EC5	Papers unrelated to the application or development of DL or ET or medical image analysis.

**Table 2:** The typical application of the ET patterns.

Application Type	ET Patterns	Mechanism	Typical Case
As weakly supervised labels	Fixations	“Image + ET” dual-channel input uses ET data as a weakly supervised label, replaces manual box selection with the observation of fixations, and trains the model to perform lesion segmentation.	A novel radiologist gaze-guided weakly supervised segmentation framework [27] demonstrates superior performance in handling class scale variations.
Multimodal alignment	Heatmaps	Align the heatmaps with the words and sentences in the radiological reports to enhance the consistency of cross-modal retrieval and diagnosis of images and text.	The ET-guided multimodal alignment (EGMA) framework [28] precisely aligns sentence-level text with image regions using gaze heatmaps.
Visual search modeling	Saccades and scan paths	Train the model using the scan paths to predict the complete search trajectory of doctors when locating lesions.	The ChestSearch model [29] reproduces the chest X-ray diagnosis pathway on the GazeSearch dataset.
Interpretability validation	Heatmaps	Compare the model’s activation map with the expert’s gaze heatmaps to verify whether the model is focusing on clinically critical areas.	By incorporating the radiologists’ ET heatmaps, we can determine whether the attention mechanism of the model is reasonable [30].

**Table 5:** Quantitative presentation of typical tasks integrated into ET-DL.

Task	Study (ET Strategy)	Modality and Disease	Dataset	Reported Metric(s) with ET	Highlights
Detection	Gaze-DETR [49]	Colposcopy (vulvovaginal candidiasis)	Colposcopy images	Average precision increased at different thresholds, and average recall increased to 0.988.	ET is encoded as sparse attention weights concatenated with image features.
Classification	RadioTransformer [50]	Chest X-ray (pneumonia)	MIMIC-CXR	F1-score ↑ and AUC ↑	By integrating visual attention into the network, the model focuses on diagnostically relevant regions of interest, leading to higher confidence in decision-making.
Annotation (auxiliary to segmentation)	ET + speech for annotation [72]	Brain MRI (brain tumor lesion marking)	BraTS	Accuracy = 92% (training), 85% (independent test)	Supports scalable, high-quality supervision for DL.

↑ Indicates an improvement in performance metrics.

**Table 6:** Application of ET in a multimodal model of medical images: strategy and performance evaluation.

Task/Domain	Multimodal Model Methods	ET Strategy	Year	Performance (Metrics)	Highlights
Radiology image classification and retrieval	EGMA [28]	Utilize radiologists’ fixation points to precisely align visual and textual elements within a dual-encoder framework.	2024	SOTA on multiple medical datasets (improved classification AUC and retrieval recall).	Gaze-guided alignment improved AUROC and retrieval through stronger image–text grounding.
Chest X-ray report generation	EGGCA-Net [105]	Integrate radiologists’ eye gaze regions (prior knowledge) to guide image–text feature alignment for report generation.	2024	Outperformed previous models on MIMIC-CXR.	ET-guided alignment produced more accurate, comprehensible radiology reports.
Chest X-ray analysis	VLMs incorporating ET data [20]	Leverage ET heatmaps overlaid on chest X-rays to highlight radiologists’ key focus areas during evaluation.	2024	Different evaluation metrics for different tasks; all the baseline models performed better with ET.	Adding gaze improved chest X-ray diagnostic accuracy across tasks.
Self-supervised medical image pre-training	GzPT [109]	Integrate ET with existing contrastive learning methods to focus on images with similar gaze patterns.	2025	SOTA on three medical datasets.	Gaze-similarity positives delivered SOTA pretraining and more interpretable features.
Knee X-ray classification	FocusContrast [110]	Use gaze to supervise the training for visual attention prediction.	2025	Consistently improved SOTA contrastive learning methods in classification accuracy.	Gaze-predicted attention consistently lifted knee X-ray classification.
Video memorability prediction	CNN + Transformer (CLIP-based spatio-temporal model) [113]	Predict memorability scores using an attention-based model aligned with human gaze fixations (collected via ET).	2025	Matched SOTA memorability prediction.	Model attention aligned with human gaze on memorable content, matching SOTA performance.
Chest radiograph abnormality diagnosis	TGID [19]	Predict radiology report intentions with temporal grounding, using fixation heatmap videos and embedded time steps as inputs.	2025	Superior to SOTA methods.	Temporal grounding from gaze improved intention detection beyond prior methods.

## Data Availability

This study’s data are publicly available.

## Abbreviations

The following abbreviations are used in this manuscript:

AI	Artificial intelligence
DL	Deep learning
ET	Eye tracking
MIA	Medical image analysis
CNNs	Convolutional neural networks
DNNs	Deep neural networks
PRISMA	Preferred Reporting Items for Systematic Reviews and Meta-Analyses
MLMs	Multimodal large models
VLMs	Vision–language models
CAD	Computer-aided diagnosis
C–CAD	Collaborative CAD
SM	Saliency map
McGIP	Medical Contrastive Gaze Image Pre-training
EGMA	Eye movement guided multimodal alignment
SOTA	State-of-the-art
ViT	Vision transformer
EG-ViT	Eye gaze guided vision transformer
SAM	Segment anything model
GCA	Gaze-conditioned augmentation
AUROC	Area Under the Receiver Operating Characteristic Curve

## References

[1] Suganyadevi S., Seethalakshmi V., Balasamy K. (2022). A review on deep learning in medical image analysis. Int. J. Multimed. Inf. Retr..

[2] Khera R., Simon M.A., Ross J.S. (2023). Automation bias and assistive AI: Risk of harm from AI-driven clinical decision support. JAMA.

[3] Mir A.N., Rizvi D.R. (2025). Advancements in deep learning and explainable artificial intelligence for enhanced medical image analysis: A comprehensive survey and future directions. Eng. Appl. Artif. Intell..

[4] Li M., Jiang Y., Zhang Y., Zhu H. (2023). Medical image analysis using deep learning algorithms. Front. Public Health.

[5] Mazurowski M.A., Dong H., Gu H., Yang J., Konz N., Zhang Y. (2023). Segment anything model for medical image analysis: An experimental study. Med. Image Anal..

[6] Sistaninejhad B., Rasi H., Nayeri P. (2023). A review paper about deep learning for medical image analysis. Comput. Math. Method Med..

[7] Rana M., Bhushan M. (2023). Machine learning and deep learning approach for medical image analysis: Diagnosis to detection. Multimed. Tools Appl..

[8] Chen X., Wang X., Zhang K., Fung K., Thai T.C., Moore K., Mannel R.S., Liu H., Zheng B., Qiu Y. (2022). Recent advances and clinical applications of deep learning in medical image analysis. Med. Image Anal..

[9] Ma C., Zhao L., Chen Y., Guo L., Zhang T., Hu X., Shen D., Jiang X., Liu T. (2024). Rectify ViT shortcut learning by visual saliency. IEEE Trans. Neural Netw. Learn. Syst..

[10] Yu K., Chen J., Ding X., Zhang D. (2025). Exploring cognitive load through neuropsychological features: An analysis using fNIRS-eye tracking. Med. Biol. Eng. Comput..

[11] Meng F., Li F., Wu S., Yang T., Xiao Z., Zhang Y., Liu Z., Lu J., Luo X. (2023). Machine learning-based early diagnosis of autism according to eye movements of real and artificial faces scanning. Front. Neurosci..

[12] Castner N., Arsiwala-Scheppach L., Mertens S., Krois J., Thaqi E., Kasneci E., Wahl S., Schwendicke F. (2024). Expert gaze as a usability indicator of medical AI decision support systems: A preliminary study. NPJ Digit. Med..

[13] Geirhos R., Jacobsen J., Michaelis C., Zemel R., Brendel W., Bethge M., Wichmann F.A. (2020). Shortcut learning in deep neural networks. Nat. Mach. Intell..

[14] Mall S., Krupinski E., Mello-Thoms C. Missed cancer and visual search of mammograms: What feature based machine-learning can tell us that deep-convolution learning cannot. Proceedings of the Medical Imaging 2019: Image Perception, Observer Performance, and Technology Assessment.

[15] Mall S., Brennan P.C., Mello-Thoms C. (2018). Modeling visual search behavior of breast radiologists using a deep convolution neural network. J. Med. Imaging.

[16] Shneiderman B. (2022). Human-Centered AI.

[17] Neves J., Hsieh C., Nobre I.B., Sousa S.C., Ouyang C., Maciel A., Duchowski A., Jorge J., Moreira C. (2024). Shedding light on ai in radiology: A systematic review and taxonomy of eye gaze-driven interpretability in deep learning. Eur. J. Radiol..

[18] Moradizeyveh S., Tabassum M., Liu S., Newport R.A., Beheshti A., Di Ieva A. (2024). When eye-tracking meets machine learning: A systematic review on applications in medical image analysis. arXiv.

[19] Awasthi A., Ahmad S., Le B., Van Nguyen H. (2025). Decoding radiologists’ intentions: A novel system for accurate region identification in chest X-ray image analysis. arXiv.

[20] Kim Y., Wu J., Abdulle Y., Gao Y., Wu H. (2024). Enhancing human-computer interaction in chest X-ray analysis using vision and language model with eye gaze patterns. arXiv.

[21] Ibragimov B., Mello-Thoms C. (2024). The use of machine learning in eye tracking studies in medical imaging: A review. IEEE J. Biomed. Health Inform..

[22] Page M.J., Mckenzie J.E., Bossuyt P.M., Boutron I., Hoffmann T.C., Mulrow C.D., Shamseer L., Tetzlaff J.M., Akl E.A., Brennan S.E. (2021). The PRISMA 2020 statement: An updated guideline for reporting systematic reviews. BMJ.

[23] Booth A., Clarke M., Dooley G., Ghersi D., Moher D., Petticrew M., Stewart L. (2012). The nuts and bolts of PROSPERO: An international prospective register of systematic reviews. Syst. Rev..

[24] Carter B.T., Luke S.G. (2020). Best practices in eye tracking research. Int. J. Psychophysiol..

[25] Dowiasch S., Wolf P., Bremmer F. (2020). Quantitative comparison of a mobile and a stationary video-based eye-tracker. Behav. Res. Methods.

[26] Kaduk T., Goeke C., Finger H., König P. (2024). Webcam eye tracking close to laboratory standards: Comparing a new webcam-based system and the EyeLink 1000. Behav. Res. Methods.

[27] Xie J., Zhang Q., Cui Z., Ma C., Zhou Y., Wang W., Shen D. (2025). Integrating eye tracking with grouped fusion networks for semantic segmentation on mammogram images. IEEE Trans. Med. Imaging.

[28] Ma C., Jiang H., Chen W., Li Y., Wu Z., Yu X., Liu Z., Guo L., Zhu D., Zhang T. Eye-gaze guided multi-modal alignment for medical representation learning. Proceedings of the 38th Conference on Neural Information Processing Systems (NeurIPS 2024).

[29] Pham T.T., Nguyen T., Ikebe Y., Awasthi A., Deng Z., Wu C.C., Nguyen H., Le N. GazeSearch: Radiology findings search benchmark. Proceedings of the IEEE/CVF Winter Conference on Applications of Computer Vision (WACV 2025).

[30] Ma C., Zhao L., Chen Y., Wang S., Guo L., Zhang T., Shen D., Jiang X., Liu T. (2023). Eye-gaze-guided vision transformer for rectifying shortcut learning. IEEE Trans. Med. Imaging.

[31] Attallah O. (2024). Skin-CAD: Explainable deep learning classification of skin cancer from dermoscopic images by feature selection of dual high-level CNNs features and transfer learning. Comput. Biol. Med..

[32] Theng D., Bhoyar K.K. (2024). Feature selection techniques for machine learning: A survey of more than two decades of research. Knowl. Inf. Syst..

[33] Yan P., Sun W., Li X., Li M., Jiang Y., Luo H. (2023). PKDN: Prior knowledge distillation network for bronchoscopy diagnosis. Comput. Biol. Med..

[34] Sarkar S., Wu T., Harwood M., Silva A.C. (2024). A transfer learning-based framework for classifying lymph node metastasis in prostate cancer patients. Biomedicines.

[35] Muksimova S., Umirzakova S., Iskhakova N., Khaitov A., Cho Y.I. (2025). Advanced convolutional neural network with attention mechanism for alzheimer’s disease classification using MRI. Comput. Biol. Med..

[36] Allogmani A.S., Mohamed R.M., Al-Shibly N.M., Ragab M. (2024). Enhanced cervical precancerous lesions detection and classification using archimedes optimization algorithm with transfer learning. Sci. Rep..

[37] Kok E.M., de Bruin A.B.H., Robben S.G.F., van Merriënboer J.J.G. (2012). Looking in the same manner but seeing it differently: Bottom-up and expertise effects in radiology. Appl. Cogn. Psychol..

[38] Kok E.M., Jarodzka H. (2017). Before your very eyes: The value and limitations of eye tracking in medical education. Med. Educ..

[39] Wang S., Ouyang X., Liu T., Wang Q., Shen D. (2022). Follow my eye: Using gaze to supervise computer-aided diagnosis. IEEE Trans. Med. Imaging.

[40] Jiang H., Hou Y., Miao H., Ye H., Gao M., Li X., Jin R., Liu J. (2023). Eye tracking based deep learning analysis for the early detection of diabetic retinopathy: A pilot study. Biomed. Signal Process. Control.

[41] Dmitriev K., Marino J., Baker K., Kaufman A.E. (2021). Visual analytics of a computer-aided diagnosis system for pancreatic lesions. IEEE Trans. Vis. Comput. Graph..

[42] Franceschiello B., Noto T.D., Bourgeois A., Murray M.M., Minier A., Pouget P., Richiardi J., Bartolomeo P., Anselmi F. (2022). Machine learning algorithms on eye tracking trajectories to classify patients with spatial neglect. Comput. Methods. Programs. Biomed..

[43] Moinak B., Shubham J., Prateek P. GazeRadar: A gaze and radiomics-guided disease localization framework. Proceedings of the Medical Image Computing and Computer Assisted Intervention—MICCAI 2022.

[44] Wang S., Zhao Z., Zhang L., Shen D., Wang Q. Crafting good views of medical images for contrastive learning via expert-level visual attention. Proceedings of the 37th Conference on Neural Information Processing Systems (NeurIPS 2023).

[45] Li X., Ding H., Yuan H., Zhang W., Pang J., Cheng G., Chen K., Liu Z., Loy C.C. (2024). Transformer-based visual segmentation: A survey. IEEE Trans. Pattern Anal. Mach. Intell..

[46] Dosovitskiy A., Beyer L., Kolesnikov A., Weissenborn D., Zhai X., Unterthiner T., Dehghani M., Minderer M., Heigold G., Gelly S. An image is worth 16×16 words: Transformers for image recognition at scale. Proceedings of the 9th International Conference on Learning Representations (ICLR 2021).

[47] He K., Gan C., Li Z., Rekik I., Yin Z., Ji W., Gao Y., Wang Q., Zhang J., Shen D. (2023). Transformers in medical image analysis. Intell. Med..

[48] Azad R., Kazerouni A., Heidari M., Aghdam E.K., Molaei A., Jia Y., Jose A., Roy R., Merhof D. (2024). Advances in medical image analysis with vision transformers: A comprehensive review. Med. Image Anal..

[49] Kong Y., Wang S., Cai J., Zhao Z., Shen Z., Li Y., Fei M., Wang Q. (2024). Gaze-DETR: Using expert gaze to reduce false positives in vulvovaginal candidiasis screening. arXiv.

[50] Bhattacharya M., Jain S., Prasanna P. RadioTransformer: A cascaded global-focal transformer for visual attention–guided disease classification. Proceedings of the 17th European Conference on Computer Vision(ECCV).

[51] Castelvecchi D. (2016). Can we open the black box of AI?. Nature.

[52] Lipton Z.C. (2018). The mythos of model interpretability. Commun. ACM.

[53] Hulsen T. (2023). Explainable artificial intelligence (XAI): Concepts and challenges in healthcare. AI.

[54] Hildt E. (2025). What is the role of explainability in medical artificial intelligence? A case-based approach. Bioengineering.

[55] Barredo Arrieta A., Díaz-Rodríguez N., Del Ser J., Bennetot A., Tabik S., Barbado A., Garcia S., Gil-Lopez S., Molina D., Benjamins R. (2020). Explainable artificial intelligence (XAI): Concepts, taxonomies, opportunities and challenges toward responsible AI. Inf. Fusion.

[56] Awasthi A., Le N., Deng Z., Agrawal R., Wu C.C., Van Nguyen H. (2024). Bridging human and machine intelligence: Reverse-engineering radiologist intentions for clinical trust and adoption. Comp. Struct. Biotechnol. J..

[57] Bhati D., Neha F., Amiruzzaman M. (2024). A survey on explainable artificial intelligence (XAI) techniques for visualizing deep learning models in medical imaging. J. Imaging.

[58] Tjoa E., Guan C. (2021). A survey on explainable artificial intelligence (XAI): Toward medical XAI. IEEE Trans. Neural Netw. Learn. Syst..

[59] Sadeghi Z., Alizadehsani R., Cifci M.A., Kausar S., Rehman R., Mahanta P., Bora P.K., Almasri A., Alkhawaldeh R.S., Hussain S. (2024). A review of explainable artificial intelligence in healthcare. Comput. Electr. Eng..

[60] Simonyan K., Vedaldi A., Zisserman A. Deep inside convolutional networks: Visualising image classification models and saliency maps. Proceedings of the 2nd International Conference on Learning Representations (ICLR 2014).

[61] Selvaraju R.R., Cogswell M., Das A., Vedantam R., Parikh D., Batra D. Grad-CAM: Visual explanations from deep networks via gradient-based localization. Proceedings of the IEEE International Conference on Computer Vision (ICCV).

[62] Smilkov D., Thorat N., Kim B., Egas F.V., Wattenberg M. SmoothGrad: Removing noise by adding noise. Proceedings of the 34th International Conference on Machine Learning (ICML) (ICML 2017).

[63] Sundararajan M., Taly A., Yan Q. Axiomatic attribution for deep networks. Proceedings of the 34th International Conference on Machine Learning (ICML 2017).

[64] Kapishnikov A., Venugopalan S., Avci B., Wedin B., Terry M., Bolukbasi T. Guided integrated gradients: An adaptive path method for removing noise. Proceedings of the IEEE/CVF Conference on Computer Vision and Pattern Recognition (CVPR).

[65] Brunyé T.T., Mercan E., Weaver D.L., Elmore J.G. (2017). Accuracy is in the eyes of the pathologist: The visual interpretive process and diagnostic accuracy with digital whole slide images. J. Biomed. Inform..

[66] Liu S., Chen W., Liu J., Luo X., Shen L. (2024). GEM: Context-aware gaze EstiMation with visual search behavior matching for chest radiograph. arXiv.

[67] Kim J., Zhou H., Lipton Z. Do you see what i see? A comparison of radiologist eye gaze to computer vision saliency maps for chest X-ray classification. Proceedings of the International Conference on Machine Learning (ICML).

[68] Khosravan N., Celik H., Turkbey B., Jones E.C., Wood B., Bagci U. (2019). A collaborative computer aided diagnosis (c-CAD) system with eye-tracking, sparse attentional model, and deep learning. Med. Image Anal..

[69] Aresta G., Ferreira C., Pedrosa J., Araujo T., Rebelo J., Negrao E., Morgado M., Alves F., Cunha A., Ramos I. (2020). Automatic lung nodule detection combined with gaze information improves radiologists’ screening performance. IEEE J. Biomed. Health Inform..

[70] Moreira I.C., Amaral I., Domingues I., Cardoso A., Cardoso M.J., Cardoso J.S. (2012). INbreast: Toward a full-field digital mammographic database. Acad. Radiol..

[71] SIIM-ACR Pneumothorax Segmentation. https://www.kaggle.com/c/siim-acr-pneumothorax-segmentation.

[72] Stember J.N., Celik H., Krupinski E., Chang P.D., Mutasa S., Wood B.J., Lignelli A., Moonis G., Schwartz L.H., Jambawalikar S. (2019). Eye tracking for deep learning segmentation using convolutional neural networks. J. Digit. Imaging.

[73] Stember J.N., Celik H., Gutman D., Swinburne N., Young R., Eskreis-Winkler S., Holodny A., Jambawalikar S., Wood B.J., Chang P.D. (2021). Integrating eye tracking and speech recognition accurately annotates MR brain images for deep learning: Proof of principle. Radiol. Artif. Intell..

[74] Khosravan N., Celik H., Turkbey B., Cheng R., Mccreedy E., Mcauliffe M., Bednarova S., Jones E., Chen X., Choyke P. Gaze2segment: A pilot study for integrating eye-tracking technology into medical image segmentation. Proceedings of the 19th International Conference on Medical Image Computing and Computer-Assisted Intervention (MICCAI 2016).

[75] Wang B., Aboah A., Zhang Z., Pan H., Bagci U. GazeSAM: Interactive image segmentation with eye gaze and segment anything model. Proceedings of the Gaze Meets Machine Learning Workshop.

[76] Colonnese F., Di Luzio F., Rosato A., Panella M. (2024). Enhancing autism detection through gaze analysis using eye tracking sensors and data attribution with distillation in deep neural networks. Sensors.

[77] Karargyris A., Kashyap S., Lourentzou I., Wu J.T., Sharma A., Tong M., Abedin S., Beymer D., Mukherjee V., Krupinski E.A. (2021). Creation and validation of a chest X-ray dataset with eye-tracking and report dictation for AI development. Sci. Data.

[78] Tian Y., Sharma A., Mehta S., Kaushal S., Liebmann J.M., Cioffi G.A., Thakoor K.A. (2024). Automated identification of clinically relevant regions in glaucoma OCT reports using expert eye tracking data and deep learning. Transl. Vis. Sci. Technol..

[79] Huang Y., Li X., Yang L., Gu L., Zhu Y., Hirofumi S., Meng Q., Harada T., Sato Y. Leveraging human selective attention for medical image analysis with limited training data. Proceedings of the 32nd British Machine Vision Conference (BMVC 2021).

[80] Zhu H., Salcudean S., Rohling R. Gaze-guided class activation mapping: Leveraging human attention for network attention in chest X-rays classification. Proceedings of the 15th International Symposium on Visual Information Communication and Interaction (VINCI 2022).

[81] Zhao Z., Wang S., Wang Q., Shen D. Mining gaze for contrastive learning toward computer-assisted diagnosis. Proceedings of the 38th AAAI Conference on Artificial Intelligence, AAAI 2024.

[82] Panetta K., Rajendran R., Ramesh A., Rao S., Agaian S. (2022). Tufts dental database: A multimodal panoramic X-ray dataset for benchmarking diagnostic systems. IEEE J. Biomed. Health Inform..

[83] Menze B.H., Jakab A., Bauer S., Kalpathy-Cramer J., Farahani K., Kirby J., Burren Y., Porz N., Slotboom J., Wiest R. (2015). The multimodal brain tumor image segmentation benchmark (BRATS). IEEE Trans. Med. Imaging.

[84] Gu J., Wang Z., Kuen J., Ma L., Shahroudy A., Shuai B., Liu T., Wang X., Wang G., Cai J. (2018). Recent advances in convolutional neural networks. Pattern Recognit..

[85] Chen P., Gao L., Shi X., Allen K., Yang L. (2019). Fully automatic knee osteoarthritis severity grading using deep neural networks with a novel ordinal loss. Comput. Med. Imaging. Graph..

[86] Rother C., Kolmogorov V., Blake A. (2004). GrabCut: Interactive foreground extraction using iterated graph cuts. ACM Trans. Graph..

[87] Martin D., Fowlkes C., Tal D., Malik J. (2001). A Database of Human Segmented Natural Images and Its Application to Evaluating Segmentation Algorithms and Measuring Ecological Statistics.

[88] Kirillov A., Mintun E., Ravi N., Mao H., Rolland C., Gustafson L., Xiao T., Whitehead S., Berg A.C., Lo W. Segment anything. Proceedings of the 20th IEEE/CVF International Conference on Computer Vision (ICCV 2023).

[89] Gutiérrez J., Che Z., Zhai G., Le Callet P. (2021). Saliency4ASD: Challenge, dataset and tools for visual attention modeling for autism spectrum disorder. Signal Process. Image Commun..

[90] Wei Q., Dong W., Yu D., Wang K., Yang T., Xiao Y., Long D., Xiong H., Chen J., Xu X. (2024). Early identification of autism spectrum disorder based on machine learning with eye-tracking data. J. Affect. Disord..

[91] Liaqat S., Wu C., Duggirala P.R., Cheung S.S., Chuah C., Ozonoff S., Young G. (2021). Predicting ASD diagnosis in children with synthetic and image-based eye gaze data. Signal Process. Image Commun..

[92] Chen S., Zhao Q. Attention-based autism spectrum disorder screening with privileged modality. Proceedings of the IEEE/CVF International Conference on Computer Vision (ICCV).

[93] Isensee F., Jaeger P.F., Kohl S.A.A., Petersen J., Maier-Hein K.H. (2021). Nnu-net: A self-configuring method for deep learning-based biomedical image segmentation. Nat. Methods.

[94] Chen C., Liu X., Ding M., Zheng J., Li J. (2019). 3d dilated multi-fiber network for real-time brain tumor segmentation in MRI. arXiv.

[95] He K., Zhang X., Ren S., Sun J. Deep residual learning for image recognition. Proceedings of the IEEE Conference on Computer Vision and Pattern Recognition (CVPR 2016).

[96] Liu Z., Lin Y., Cao Y., Hu H., Wei Y., Zhang Z., Lin S., Guo B. Swin transformer: Hierarchical vision transformer using shifted windows. Proceedings of the IEEE/CVF International Conference on Computer Vision (ICCV 2021).

[97] Tan M., Le Q.V. EfficientNet: Rethinking model scaling for convolutional neural networks. Proceedings of the 36th International Conference on Machine Learning (ICML 2019).

[98] Leveque L., Bosmans H., Cockmartin L., Liu H. (2018). State of the art: Eye-tracking studies in medical imaging. IEEE Access.

[99] Zuo F., Jing P., Sun J., Duan J., Ji Y., Liu Y. (2024). Deep learning-based eye-tracking analysis for diagnosis of alzheimer’s disease using 3d comprehensive visual stimuli. IEEE J. Biomed. Health Inform..

[100] Kumar S., Rani S., Sharma S., Min H. (2024). Multimodality fusion aspects of medical diagnosis: A comprehensive review. Bioengineering.

[101] Baltrusaitis T., Ahuja C., Morency L. (2019). Multimodal machine learning: A survey and taxonomy. IEEE Trans. Pattern Anal. Mach. Intell..

[102] Warner E., Lee J., Hsu W., Syeda-Mahmood T., Kahn C.E., Gevaert O., Rao A. (2024). Multimodal machine learning in image-based and clinical biomedicine: Survey and prospects. Int. J. Comput. Vis..

[103] Kabir R., Haque N., Islam M.S., Marium-E-Jannat (2024). A comprehensive survey on visual question answering datasets and algorithms. arXiv.

[104] Masse B., Ba S., Horaud R. (2018). Tracking gaze and visual focus of attention of people involved in social interaction. IEEE Trans. Pattern Anal. Mach. Intell..

[105] Peng P., Fan W., Shen Y., Liu W., Yang X., Zhang Q., Wei X., Zhou D. (2024). Eye gaze guided cross-modal alignment network for radiology report generation. IEEE J. Biomed. Health Inform..

[106] Lanfredi R.B., Zhang M., Auffermann W.F., Chan J., Duong P.T., Srikumar V., Drew T., Schroeder J.D., Tasdizen T. (2022). REFLACX, a dataset of reports and eye-tracking data for localization of abnormalities in chest X-rays. Sci. Data.

[107] MIMIC-Eye: Integrating MIMIC Datasets with REFLACX and Eye Gaze for Multimodal Deep Learning Applications. https://physionet.org/content/mimic-eye-multimodal-datasets/1.0.0/.

[108] Tutek M., Šnajder J. (2022). Toward practical usage of the attention mechanism as a tool for interpretability. IEEE Access.

[109] Wang S., Zhao Z., Shen Z., Wang B., Wang Q., Shen D. (2025). Improving self-supervised medical image pre-training by early alignment with human eye gaze information. IEEE Trans. Med. Imaging.

[110] Wang S., Zhao Z., Zhuang Z., Ouyang X., Zhang L., Li Z., Ma C., Liu T., Shen D., Wang Q. (2025). Learning better contrastive view from radiologist’s gaze. Pattern Recognit..

[111] Zeger E., Pilanci M. (2024). Black boxes and looking glasses: Multilevel symmetries, reflection planes, and convex optimization in deep networks. arXiv.

[112] Zhang M., Cui Q., Lü Y., Yu W., Li W. (2024). A multimodal learning machine framework for alzheimer’s disease diagnosis based on neuropsychological and neuroimaging data. Comput. Ind. Eng..

[113] Kumar P., Khandelwal E., Tapaswi M., Sreekumar V. (2025). Seeing eye to AI: Comparing human gaze and model attention in video memorability. arXiv.

[114] Khosravi S., Khan A.R., Zoha A., Ghannam R. Self-directed learning using eye-tracking: A comparison between wearable head-worn and webcam-based technologies. Proceedings of the IEEE Global Engineering Education Conference (EDUCON).

[115] Kora P., Ooi C.P., Faust O., Raghavendra U., Gudigar A., Chan W.Y., Meenakshi K., Swaraja K., Plawiak P., Rajendra Acharya U. (2022). Transfer learning techniques for medical image analysis: A review. Biocybern. Biomed. Eng..

[116] Saxena S., Fink L.K., Lange E.B. (2024). Deep learning models for webcam eye tracking in online experiments. Behav. Res. Methods.

[117] Valtakari N.V., Hooge I.T.C., Viktorsson C., Nyström P., Falck-Ytter T., Hessels R.S. (2021). Eye tracking in human interaction: Possibilities and limitations. Behav. Res. Methods.

[118] Sharafi Z., Sharif B., Guéhéneuc Y., Begel A., Bednarik R., Crosby M. (2020). A practical guide on conducting eye tracking studies in software engineering. Empir. Softw. Eng..

[119] Guideline for Reporting Standards of Eye-Tracking Research in Decision Sciences. https://osf.io/preprints/psyarxiv/f6qcy_v1.

[120] Seyedi S., Jiang Z., Levey A., Clifford G.D. (2022). An investigation of privacy preservation in deep learning-based eye-tracking. Biomed. Eng. Online.

